# Evidence for SARS-CoV-2 related coronaviruses circulating in bats and pangolins in Southeast Asia

**DOI:** 10.1038/s41467-021-21240-1

**Published:** 2021-02-09

**Authors:** Supaporn Wacharapluesadee, Chee Wah Tan, Patarapol Maneeorn, Prateep Duengkae, Feng Zhu, Yutthana Joyjinda, Thongchai Kaewpom, Wan Ni Chia, Weenassarin Ampoot, Beng Lee Lim, Kanthita Worachotsueptrakun, Vivian Chih-Wei Chen, Nutthinee Sirichan, Chanida Ruchisrisarod, Apaporn Rodpan, Kirana Noradechanon, Thanawadee Phaichana, Niran Jantarat, Boonchu Thongnumchaima, Changchun Tu, Gary Crameri, Martha M. Stokes, Thiravat Hemachudha, Lin-Fa Wang

**Affiliations:** 1 Thai Red Cross Emerging Infectious Diseases Health Science Centre, WHO Collaborating Centre for Research and Training on Viral Zoonoses, King Chulalongkorn Memorial Hospital, Faculty of Medicine, Chulalongkorn University, Bangkok, Thailand; 2 Programme in Emerging Infectious Diseases, Duke-NUS Medical School, Singapore, Singapore; 3 Department of National Parks, Wildlife and Plant Conservation, Ministry of Natural Resources and Environment, Bangkok, Thailand; 4 Forest Biology Department, Faculty of Forestry, Kasetsart University, Bangkok, Thailand; 5 Changchun Veterinary Research Institute, Chinese Academy of Agricultural Sciences, Changchun, China; 6 Jiangsu Co-innovation Center for Prevention and Control of Important Animal Infectious Diseases and Zoonosis, Yangzhou University, Yangzhou, China; 7 CSIRO Australian Animal Health Laboratory, Geelong, Australia; 8 Biological Threat Reduction Program, Defense Threat Reduction Agency, Virginia, USA; 9 SingHealth Duke-NUS Global Health Institute, Singapore, Singapore

**Keywords:** Pathogens, SARS-CoV-2

## Abstract

Among the many questions unanswered for the COVID-19 pandemic are the origin of SARS-CoV-2 and the potential role of intermediate animal host(s) in the early animal-to-human transmission. The discovery of RaTG13 bat coronavirus in China suggested a high probability of a bat origin. Here we report molecular and serological evidence of SARS-CoV-2 related coronaviruses (SC2r-CoVs) actively circulating in bats in Southeast Asia. Whole genome sequences were obtained from five independent bats (*Rhinolophus acuminatus*) in a Thai cave yielding a single isolate (named RacCS203) which is most related to the RmYN02 isolate found in *Rhinolophus malayanus* in Yunnan, China. SARS-CoV-2 neutralizing antibodies were also detected in bats of the same colony and in a pangolin at a wildlife checkpoint in Southern Thailand. Antisera raised against the receptor binding domain (RBD) of RmYN02 was able to cross-neutralize SARS-CoV-2 despite the fact that the RBD of RacCS203 or RmYN02 failed to bind ACE2. Although the origin of the virus remains unresolved, our study extended the geographic distribution of genetically diverse SC2r-CoVs from Japan and China to Thailand over a 4800-km range. Cross-border surveillance is urgently needed to find the immediate progenitor virus of SARS-CoV-2.

## Abstract (web summary)

A bat origin for SARS-CoV-2 has been proposed. Here, by sampling wild *Rhinolophus acuminatus* bats from Thailand, the authors identified a SARS-CoV-2-related coronavirus (SC2r-CoV), designated as RacCS203, with 91.5% genome similarity to SARS-CoV-2, and show that sera obtained from bats and Malayan pangolin neutralize SARS-CoV-2.

## Introduction

The coronavirus disease 2019 (COVID-19) pandemic^1^ was caused by a previous unknown coronavirus, named severe acute respiratory syndrome coronavirus 2 (SARS-CoV-2)^2,3^. In less than a year, the COVID-19 pandemic claimed more than 1.6 million lives and SARS-CoV-2 infected more than 75 million people all around the world^4^. In addition to the health and economic challenges COVID-19 has posed, there are also many scientific challenges identified at the different stages of the pandemic with several key scientific questions remain unanswered^5,6^. One of them is the origin of SARS-CoV-2 and its early transmission event from animal to human^6^.

Taxonomically, SARS-CoV-2 is classified as a member of the species *SARS-related coronavirus* (SARSr-CoV) in the genus *Betacoronavirus* of the family *Coronaviridae*^3^. An early report by Zhou et al. identified a closely related SARSr-CoV genome sequence, RaTG13, which shared a 96% whole-genome sequence identity with SARS-CoV-2, indicating a probable bat origin of SARS-CoV-2^2^. Since then, more SARS-CoV-2-related viral genome sequences from bats have been reported from Eastern China^7^ and Japan^8^, and from pangolins in China^9,10^. However, the immediate animal ancestor or progenitor virus, the equivalent of the >99% identical SARS-CoV sequences identified in civets during the SARS outbreak in 2003^11^, remains elusive for SARS-CoV-2. Identification of the origin and immediate progenitor viruses are not only important academically, but also critical for public health measures to prevent future outbreaks caused by SARS-CoV-2 or closely related viruses^12–14^.

Here, we conducted surveillance investigations in Southeast Asia focusing on bats and pangolins as SARS-CoV-2-related viruses have previously been detected in these animals^9,10,15^. While PCR- and sequencing-based methods provide more conclusive data, past experience in searching for virus origins has demonstrated that serological surveys can be superior and have a higher chance of success due to the fact that virus-specific antibodies last much longer than viral genetic material in infected animals^16–18^.

## Results

### Bats sampling in Chachoengsao Province, Thailand

**Fig. 1:**
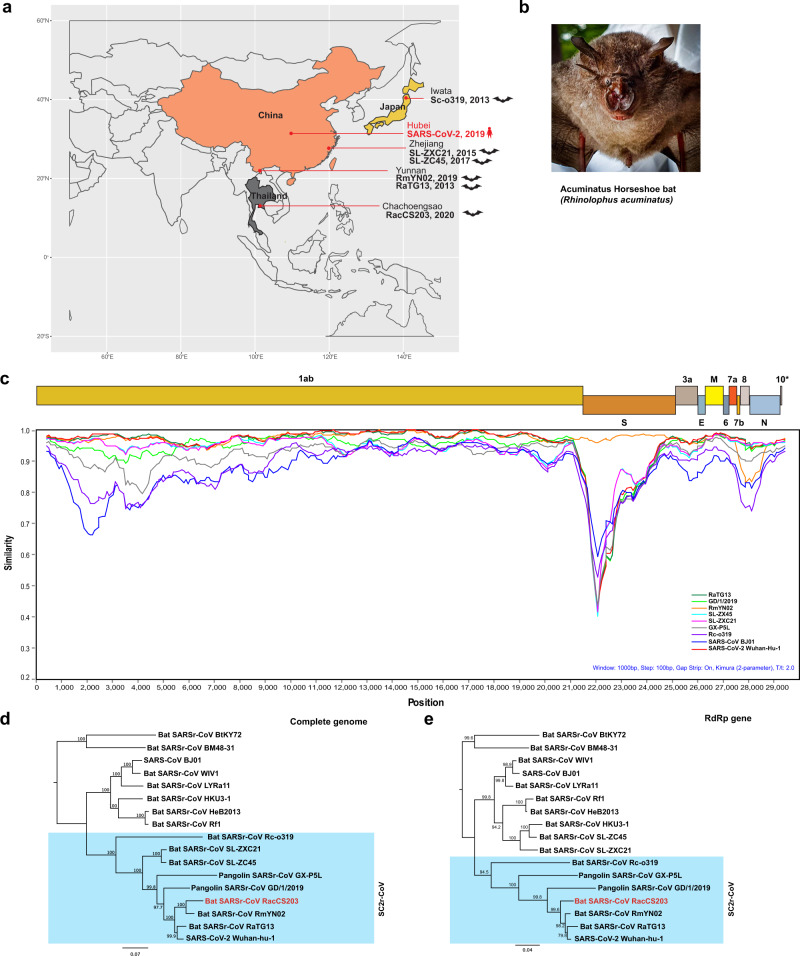
Molecular detection of a SC2r-CoV in bats in Thailand. **a** Map of Asia illustrating the SC2r-CoVs detected in this region to date. **b** The Acuminatus horseshoe bats from which the SC2r-CoV was detected. Photo taken by the Thai research team of this study group. **c** Similarity plot (SimPlot) of whole-genome sequences of 10 SARSr-CoVs using the RacCS203 as a reference genome. **d** Phylogenetic tree based on whole-genome sequences. **e** Phylogenetic tree based on the RdRp gene sequences. The trees in **d** and **e** were generated using PhyML with general-time-reversible (GTR) substitution model and 1000 bootstrap replicates. Numbers (>70) above or below the branches are percentage bootstrap values for the associated nodes. The scale bar represents the number of substitutions per site. RacCS203 was highlighted in red.

A colony of ~300 bats was located in an artificial cave of a 1-m-diameter irrigation water pipe in a Wildlife Sanctuary, Chachoengsao (CS) Province of Eastern Thailand (Fig. 1a). A total of 100 bats were captured and sampled in June 2020 (Supplementary Table 1). There was only one species of bat found at the site, which was identified as *Rhinolophus acuminatus* by morphology (Fig. 1b) and DNA sequencing of the cytochrome c oxidase subunit I (COI) gene.

### Phylogenetic analysis of the SC2r-CoV RacCS203

When screened by a published pan-CoV PCR method targeting a 328-bp region in the RdRp gene^19^, 13 of the 100 rectal swabs were positive (Supplementary Table 1). Sequencing of PCR amplicons from all positive samples revealed an identical sequence which has the highest sequence identity of 96.21% to bat CoV-RaTG13 (NCBI accession no. MN996532.1), 95.86% to human SARS-CoV-2 (NCBI accession no. MT631834.1), and 94.48% to CoV-RmYN02 (GISAID no. EPI_ISL_412977). Five samples with high levels of viral RNA were chosen for further analysis by next-generation sequencing (NGS). The whole-genome sequence with the best assembly quality, named RacCS203, was used as a reference genome for subsequent analysis. The sequences from the other four genomes were almost identical to RacCS203 (Supplementary Fig. 1), indicating this to be the most dominant CoV circulating in this bat colony at the time of sampling. Similarity plot of the whole genome (Fig. 1c) and comparison of deduced protein sequences (Supplementary Tables 2 and 3) indicated that this CoV is most similar to the bat CoV, RmYN02 (ref. ^7^), with genome sequence identity of 93.7%. Phylogenetic analyses based on the whole genome (Fig. 1d) and the RdRp gene (Fig. 1e) placed RacCS203 as a new member of the SARS-CoV-2-related coronavirus (SC2r-CoV) lineage. For easier comparison and to avoid confusion, we have named the SARS-CoV-related coronaviruses as SC1r-CoV lineage in this paper. Despite the close relatedness between RacCS203 and RmYN02, there are multiple molecular features indicating that RacCS203 is sufficiently different from RmYN02 and hence a novel bat CoV. First, from the similarity plot presented in Fig. 1c, it is clear that at the genome level, these two viruses differ at multiple regions in the ORF1ab gene and with major difference toward the 3ʹ end of the genome covering ORFs 7, 8, and part of the N gene. Second, overall aa sequence identity of ortholog proteins also differs from protein to protein (Supplementary Tables 2 and 3). Third, with a close examination of aa sequences, it was found that the ORF8 has a higher sequence identity to that of ZC45 than RmYN02 (29.8% amino acid sequence identity) (Supplementary Fig. 2). On the other hand, the ORF10 proteins of ZC45 and RmYN02 were identical whereas the RacCS203 ORF10 was significantly different from these two, but identical to that of ZXC21.

### Analysis of RacCS203 receptor binding domain and function

**Fig. 2:**
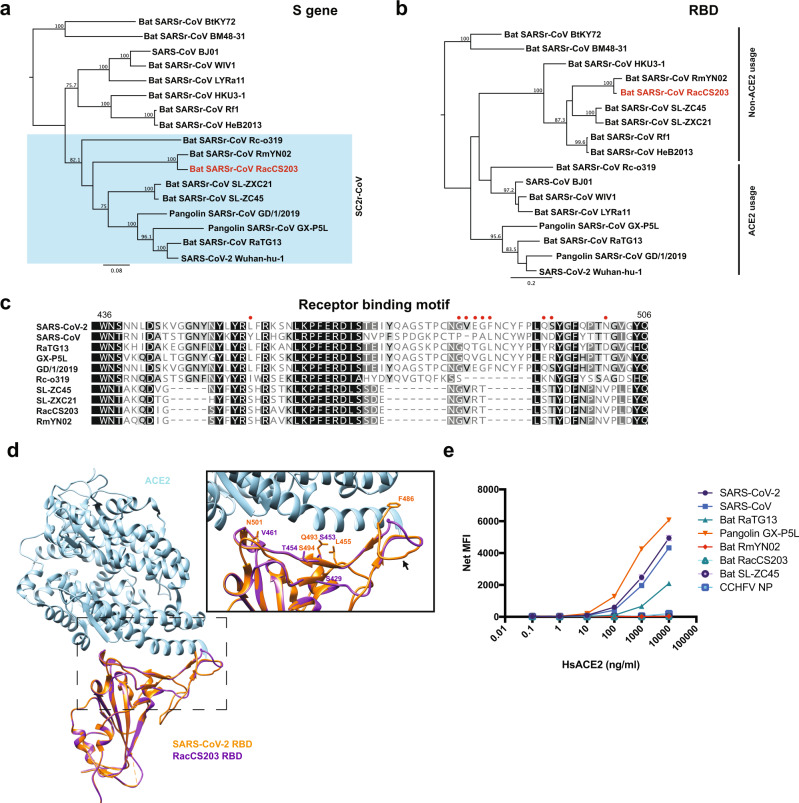
Analysis of RBD sequences and function. **a** Phylogenetic tree based on the S gene sequences. **b** Phylogenetic tree based on the nucleotide sequences of the RBD coding regions. The trees in **a** and **b** were generated using PhyML with substitution model GTR and 1000 bootstraps. Numbers (>70) above or below the branches are percentage bootstrap values for the associated nodes. The scale bar represents the number of substitutions per site. **c** Amino acid sequence comparison of the receptor binding motif (RBM) of seven SARSr-CoVs with the red dots indicating amino acid residues critical for ACE2 interaction. **d** Structural modeling of the RBD-hACE2 interaction for RacCS203 based on the known structure of the SARS-CoV-2 RBD. **e** Multiplex binding assay to measure affinity of different SARSr-CoV RBDs to PE-conjugated hACE2. Data presented were derived from three independent experiments. Error bars indicate standard deviation. Graph was plotted using GraphPad Prism 8.

We next examined the receptor binding domain (RBD) of RacCS203 at sequence, structure, and function levels. When the full-length S gene sequences were used for phylogenetic analysis, RacCS203 was grouped with other SC2r-CoVs (Fig. 2a). However, when the nucleotide sequences of the RBD coding regions were compared, RacCS203 was grouped with the non-ACE2-usage SARSr-CoVs (Fig. 2b). Data from sequence alignment focusing on the receptor binding motif (Fig. 2c) and structural modeling (Fig. 2d) are consistent with the phylogenetic analysis, indicating that the RacCS203 virus is unlikely to use ACE2 as an entry receptor. This was experimentally confirmed by RBD-ACE2 binding studies conducted using purified recombinant proteins. Using a multiplex RBD-ACE2 binding analysis assay, the different SC2r-CoV RBDs can be grouped into ACE2-usage (SARS-CoV2, RaTG13, GX-P5L) and non-ACE2-usage group (RmYN02, ZC45, and RacCS203) (Fig. 2e). To exclude the possibility that the ACE2 binding of RBD may not represent the functionality of the full-length S protein, we also constructed a VSV-based pseudovirus using previously published method^20^ with the SARS-CoV-2 S gene as a positive control. While the SARS-CoV-2 S typed pseudotyped VSV was able to infect VeroE6, the RacCS203 S typed pseudovirus failed to do so. As the sampling was done using lysis buffer in this study, no virus isolation was conducted. However, we will be attempting virus isolation in future sampling studies.

### Serological investigation

**Fig. 3:**
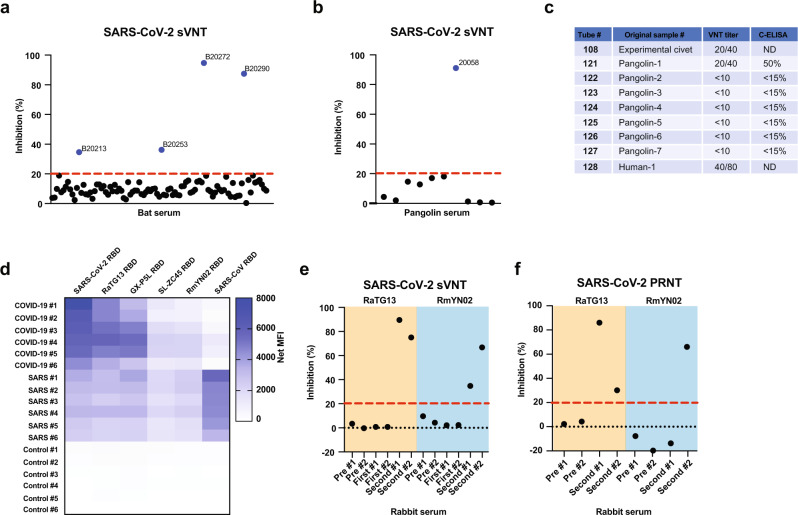
Serological investigation. **a** SARS-CoV-2 sVNT results from bat sera in Thailand. **b** SARS-CoV-2 sVNT results from pangolin sera in Thailand. **c** SARS-CoV VNT and C-ELISA testing for pangolin sera sampled from animals originated from Southeast Asia. **d** Multiplex-RBD binding study to examine antigenic cross reactivity between human sera and different RBDs. **e** SARS-CoV-2 sVNT assay to examine cross-neutralization ability of different rabbit anti-RBD sera. Data indicate percentage of inhibition at 1:20 dilution. **f** SARS-CoV-2 PRNT assay to examine cross-neutralization ability of different rabbit anti-RBD sera. Data indicate percentage of inhibition at 1:10 dilution. Red dot line indicates cut-off at 20% inhibition. Pre indicates pre-immunized sera; First and Second indicates sera obtained post first immunization and second immunization, respectively. All graphs and heatmap presented were generated using GraphPad Prism 8.

In parallel, a serological investigation was also conducted to investigate the circulation of SC2r-CoV in Southeast Asia. To achieve the highest specificity possible without having to rely on a BSL3 facility and live virus, the SARS-CoV-2 surrogate virus neutralization test (sVNT)^20^, which measures antibody-mediated inhibition of SARS-CoV-2 RBD-ACE2 interaction, was used in this study. Among the 98 serum samples examined, four showed positive virus neutralization antibodies with two showing very strong inhibition at 88% and 97%, respectively, in the sVNT assay (Fig. 3a and Supplementary Table 1).

We also tested ten pangolin sera sampled from February to July 2020 from three Wildlife Checkpoint stations in Central and Southern Thailand with unknown country origin as those were confiscated from illegal traders and quarantined under the Wild Animal Reservation and Protection Act, B.E. 2535. Sequencing of mitochondrial genes (*coi* and *cytB*)^21^ confirmed the species identification as *Manis javanica*, which is the most abundant pangolin species in this region^22^. One pangolin sample had a strong sVNT reading at 91% (Fig. 3b). This is interesting as we have historic data in China from May 2003 showing that one of seven pangolin sera were positive for SARS-CoV by both competition ELISA and VNT (Fig. 3c). Those pangolins were confiscated and quarantined at the Guangdong Wildlife Conservation and Protection Center, which were illegally smuggled into China from Southeast Asia of unknown country origin. These serology data corroborated published findings that pangolins are susceptible to infection by SC2r-CoVs^9,10,15^. PCR testing using pan-CoV primers was conducted on the limited pangolin samples in both studies and were all negative.

The RacCS203 S gene is most similar to that of RmYN02 (Supplementary Fig. 3a). The two viruses shared part of the furin cleavage site unique to SARS-CoV-2 (Supplementary Fig. 3b) and have an almost identical RBD aa sequence with only two residue differences out of 204 aa residues (Supplementary Fig. 3c). The two RBDs also share a very similar predicted 3-D structure (Supplementary Fig. 3d). As various reagents derived from RmYN02 RBD were already available in our group before the discovery of RacCS203, they were used as a surrogate for RacCS203 in some of the antigenic studies. To optimize the comparative antigenic study, we custom manufactured recombinant RBD containing biotin and 8X His tag at the C-terminus to coat the RBD onto avidin Luminex beads with fixed orientation and normalize the antigen load with anti-His monoclonal antibody quantification. When different RBDs were tested for reactivity against COVID-19 patient sera, three distinctive groups were identified. The strongest reactors were SARS-CoV-2, RaTG13, and GX-P5L, followed by the weak reactors ZC45 and RmYN02 with the SARS-CoV RBD showing the least cross reactivity. Vice versa, when SARS patient sera were used, they showed low cross reactivity against all SC2r-CoV RBDs (Fig. 3d).

To further investigate the antigenic relationship among SC2r-CoVs, rabbit sera raised against different RBDs were produced and their cross reactivity and cross neutralizing ability were examined. Notwithstanding some variation from rabbit to rabbit, the overall pattern of reactivity is as expected from the RBD sequences with the anti-RaTG13 sera having stronger cross reactivity toward SARS-CoV-2 than those raised against the RmYN02 RBD. Rabbit sera against both RBDs were able to cross neutralize SARS-CoV-2 in two different assays, the sVNT (Fig. 3e) and PRNT (Fig. 3f).

### Geographical distribution of *Rhinolophus* species

**Fig. 4:**
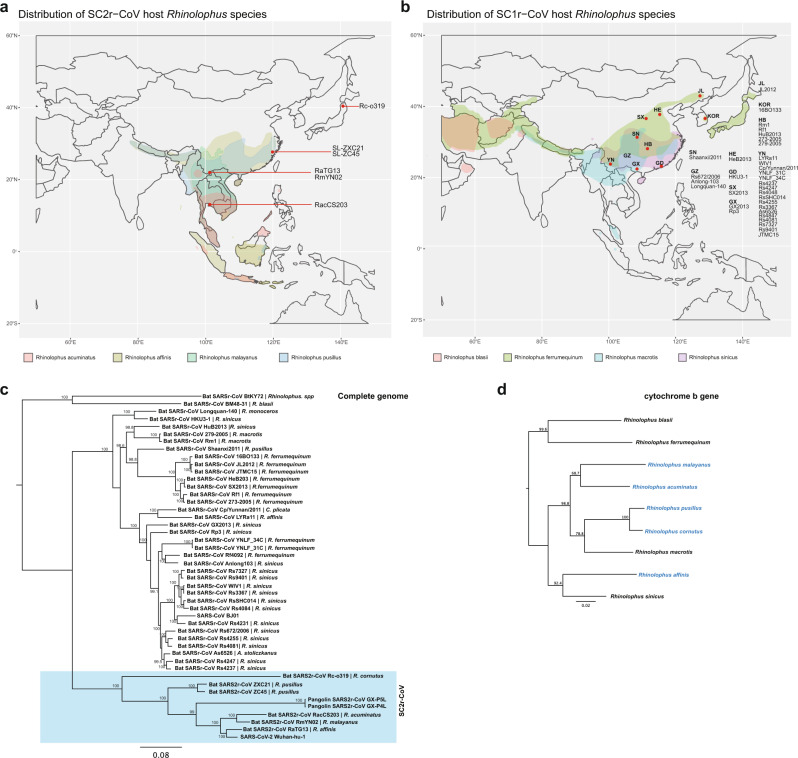
Location distribution of Rhinolophus species carrying SC1r-CoV and SC2r-CoV. Distribution of **a** SC2r-CoV and **b** SC1r-CoV in Southeast Asia; Phylogeny trees of **c** SARSr-CoVs and **d** Rhinolophus species constructed using PhyML with substitution model GTR and 1000 bootstraps. Numbers (>70) above or below the branches are percentage bootstrap values for the associated nodes. The scale bar represents the number of substitutions per site.

Finally, we examined the relationship among the phylogeny of viruses and the phylogeny and geographic locations of the different *Rhinolophus* bats known to carry SC2r-CoVs versus those for SC1r-CoVs. The SC2r-CoV-carrying bats (Fig. 4a) seem to be distributed at a lower latitude than those carrying the SC1r-CoVs (Fig. 4b) with some overlap in Southern China and the top end of the neighboring Southeast Asian nations. While the virus phylogeny can clearly separate the two lineages into SC2r-CoV and SC1r-CoV (Fig. 4c), the bat phylogeny does not have a clear evolutional distinction of the two groups (Fig. 4d). More research is needed to understand the potential species-specific distribution of different SARSr-CoVs among different bats in this part of the world.

## Discussion

In this study, we provided both molecular and serological evidence indicating that SC2r-CoVs are in circulation in bats in Southeast Asia. Although the sampling site (Thailand only) and sampling size was limited, we are confident that CoVs with a high degree of genetic relatedness to SARS-CoV-2 are widely present in bats across many nations and regions in Asia. As shown in Fig. 1a, SC2r-CoVs have now been detected in bats from Iwata Prefecture in Japan in the north, Zhejiang in the east to Yunnan in the southwest of China and southward to Chachoengsao, Thailand. This covers an area from 34.42000°N 137.506000°E to 13.409845054231498°N 101.88015941162523°E, spanning a direct distance of ~4800 km. Rhinolophus bats have a wide distribution from Australia to Europe (Supplementary Fig. 4a) and at least 22 Rhinolophus species have been recorded by Department of National Parks, Wildlife and Plant Conservation in Thailand. So far five Rhinolophus species in Asia have been shown to carry SC2r-CoVs (Supplementary Fig. 4b), and it is the first time that SC2r-CoV was identified in *R. acuminatus*. In this study, we only sampled 100 bats of one species. It is safe to predict that many more SC2r-CoVs will be discovered in bats of different Rhinolophus species in this region once surveillance studies are intensified through broader and well-coordinated international collaborations as recently outlined in a WHO report^13^.

Our data demonstrated that among the SARSr-CoVs, two lineages can be defined as SC2r-CoV and SC1r-CoVs, based on whole-genome phylogeny (Fig. 1d). However, when the RBD regions were examined, two different lineages can be defined as ACE2-usage vs non-ACE2-usage, respectively (Fig. 2b). This was functionally confirmed by RBD-ACE2 binding studies in vitro (Fig. 2e), revealing the following human ACE2 binding affinity ranking: GX-P5L>SARS-CoV-2>SARS-CoV>RaTG13. It is interesting to note that the only bat CoV hACE2-binding RBD included in this study (RaTG13) ranked the lowest and the Malayan pangolin CoV-derived RBD (GX-P5L) ranked the highest. The fact that the ACE2 usage lineages do not exactly match the two genome sequence lineages may suggest either these viruses have undergone recombination at the RBD region or ACE2 is not the only or main entry receptor used by these viruses in bats. Further investigation is needed to clarify this interesting finding.

As for our previous investigation into the origin of emerging zoonotic viruses, we demonstrated once again that serology can play a pivotal role in complementing molecular detection. In the past, serology investigation involves an initial screening with ELISA, followed by confirmation using VNT^16^. In this study, we used the newly developed sVNT platform^20^ to achieve both screening and determining neutralizing antibodies in the same test. The species-independent performance has previously been demonstrated with sera from rabbits and mice in our group^20^ and from cats, dogs, and hamsters in the University of Hong Kong group^23^. The sVNT platform has also been granted the Emergency Use Authorization by the US FDA to determine SARS-CoV-2-neutralizing antibodies in human sera, due to its good performance concordance with live virus-based VNT assays^24^.

The finding that RacCS203 RBD failed to bind hACE2 raised an interesting question on the mechanism of SARS-CoV-2 neutralizing antibodies detected in four of the 98 bats tested in this study. There are at least two possibilities. First, despite the failure in hACE2 binding, the sequence homology between the RBDs of RacCS203 RBD and SARS-CoV-2 will be sufficient to induce cross-neutralizing antibodies. This is supported by results with the rabbit anti-RmYN02 RBD sera (Fig. 3e, f). Second, there are other SC2r-CoV circulating in these bats and the neutralizing antibodies reflect past infection(s) by another CoV(s) which may be genetically more closely related to SARS-CoV-2. This is supported by the fact that we observed a higher PCR positive rate than SARS-CoV-2 sVNT positive rate in the colony and the two bat samples with the highest sVNT inhibition (BT20272 at 94.7% and BT20290 at 87.4%) were both positive by PCR with RacCS203 (Supplementary Table 1). It should be emphasized that sVNT inhibition between 87% and 94% is considered very high even for confirmed COVID-19 patients^20^ and higher than the rabbit sera immunized with the RmYN02 RBD (40–70%, Fig. 3e). It is also worth noting that bat antibodies usually have lower virus neutralization titers than those from human and other spillover mammals^25,26^. More studies are clearly needed to investigate these two possibilities.

Finally, we presented serological data supporting the notion that pangolins are susceptible to SARSr-CoV infection. Despite the very low number of animals sampled as both studies (2003 in China and 2020 in Thailand) used opportunistic sampling from confiscated animals in quarantine centers, we were able to demonstrate highly specific neutralizing antibodies to SARS-CoV (in 2003) and SARS-CoV-2 (in 2020). These findings are consistent with the molecular and serological data reported by other groups recently^9,10,15^. The fact that serum samples taken in 2003 and 2020 showed neutralizing antibodies against SARSr-CoVs implied that such viruses have been circulating in pangolins in Southeast Asia for at least 17 years. However, the current study is unable to differentiate the role of pangolins as a spillover host from a secondary reservoir host. Further investigation with more systematic and longitudinal sampling of animals in their natural habitat is required to better understand the role of pangolins in transmitting and/or maintaining SARSr-CoVs.

In conclusion, the current study provides further experimental evidence to support the notion that the distribution of SC2r-CoVs is not limited to China. Southeast Asia, due to its richness in both relevant bat species diversity and population density, may be more likely to be a hotspot for such viruses^27^. Our data also demonstrated the great genetic diversity and plasticity of the SC2r-CoV genomes. From the limited number of members (9 in total) of this lineage with published whole-genome sequences, they already display many differences that are unique to each of the members. The rich diversity of SC2r-CoVs in the region suggests that there is a high probability to find the immediate progenitor virus of SARS-CoV-2 with intensified and internationally coordinated surveillance.

## Methods

### Animal sampling

Acuminate Horseshoe bats (*R. acuminatus*) were captured from a Wildlife Sanctuary in the Chachoengsao Province, Thailand. Bats were released after measurements and samples were collected. Bats were identified morphometrically and species, sex, reproductive status, forearm length, and body mass were determined. Rectal swab and blood were collected from each individual bat and immediately put into Lysis buffer (bioMérieux, SA, France). The samples were transported on ice within 48 h and stored at −80 °C until further analysis at the Thai Red Cross Emerging Infectious Disease Health Science Centre (TRC-EID) Thailand. Sampling was performed under protocols approved and permitted by the Department of National Parks, Wildlife and Plant Conservation, Thailand (No. 0909.204/2686) and the Animal Use Protocol No. 1473001 approved by Chulalongkorn University Animal Care and Use Committee. Malayan pangolin (*M. javanica*) samples were collected between February and July 2020 from three Wildlife Checkpoint stations, Department of National Parks, Wildlife and Plant Conservation in Central and Southern Thailand with unknown country origin and sent to TRC-EID laboratory in Bangkok within 24 h for testing. For the investigation of pangolins in China, samples were collected from the Guangdong Wildlife Conservation and Protection Center with special permit issued by the National SARS Research Coordination Committee.

### Nucleic acid extraction and CoV RNA detection

Total nucleic acid was extracted from 1 ml of suspended rectal swab using easyMAG® platform (bioMérieux, SA, France) and eluted in 50 µl. Hemi-nested Reverse Transcription PCR (RT-PCR) was performed using PCR with broadly reactive consensus PCR primers targeting the RNA-dependent RNA polymerase (RdRp) gene of different CoVs (Supplementary Table 4)^19,28^. Synthesis of cDNA was carried out using the Superscript III First Strand Synthesis kit (Invitrogen) followed by nested PCR. Amplification product of 328 and 434 bp, respectively, was visualized using 2% agarose gel electrophoresis. All positive PCR products were further sequenced for confirmation and strain characterization. The RdRp PCR products were gel purified using the NucleoSpin® Gel and PCR Clean-up kit (MACHEREY-NAGEL GmbH & Co. KG) and sequenced directly using an automated ABI PRISM 377 DNA Sequencer.

### Genome characterization by NGS

Whole-genome sequencing (WGS) using NGS technology was performed on five nucleic acid specimens with relatively strong PCR positive signals. WGS was performed using enrichment library preparation (Respiratory Viral Oligos Panel, RVOP) and an Illumina MiSeq 3000 sequencer, according to the manufacturer’s instructions. Raw reads were first imported into Geneious Prime (version 2020.2.3) for downstream analysis and trimmed of adapters with BBDuk (version 38.84). De novo assembly was conducted with clean reads by SPAdes (version 3.13.0, http://cab.spbu.ru/software/spades/) in Metagenome mode. The longest contig for each sample was then blasted against SARS-CoV-2 reference genome (MN908947) to evaluate the completeness of the genome. The name RacCS203 was assigned to the best contig (29,853 nt). Each sample was then individually mapped to the reference RacCS203 genome using Geneious assembler. Coverage map, low coverage and Variant/SNP was further analyzed in Geneious. Annotation of RacCS203 was done by comparing and transferring the annotation of human SARS-CoV-2 and other related CoVs (RaTG13, BJ01, GX-P4L, SL-ZXC21, SL-ZC45, and RmYN02) after nucleotide sequence alignment done by MAFFT in Geneious Prime software. Individual gene alignment was generated by Geneious alignment and used to plot the phylogeny tree by the maximum-likelihood method with the general-time-reversible (GTR) model and 1000 bootstrap replicates in PHYML 3.0 software. Similarity plot was generated by SimPlot (version 3.5.1). The accession number of the genome sequences used in the phylogeny analysis are tabulated in Supplementary Table 5.

### Structure modeling

The three-dimension structures of RacCS203 and RmYN02 were modeled using Swiss-Model program (https://swissmodel.expasy.org/)^29^ using SARS-CoV-2 RBD-ACE2 structure (PBD accession number: 6VW1) as a template. The structures were built using UCSF Chimera 1.14.

### Antibody tests

sVNT was performed using cPass (Genscript) according to the manufacturer’s instructions. Briefly, equal volume of 1:10 diluted bat or pangolin sera were pre-incubated with HRP-conjugated RBD for 30 min at 37 °C, followed by addition to ACE2-coated ELISA plate for 15 min at room temperature. Colorimetric signal was developed using TMB substrate and stopped with 1 M HCl. Absorbance readings at 450 nm were acquired using a microplate reader. PRNT was performed by pre-incubation of 50 PFU of SARS-CoV-2 (BetaCoV/Singapore/2/2020, GISAID accession code EPI_ISL_406973), with four-fold serial diluted sera for 1 h at 37 °C, followed by infection of monolayer VeroE6 (ATCC, CRL-1586) for 1 h at 37 °C. After 1 h incubation, the inoculum was removed and the cells were overlaid with plaque medium containing 0.5% carboxylmethylcellulose and 0.5% avicel. At 3 dpi, the cells were fixed with 4% formaldehyde and the plaques were visualized following 0.2% crystal violet staining. For detection of SARS-CoV-specific antibody in the pangolin serum samples in China, a commercial competition ELISA kit (BGI-GBI) was used following manufacturer’s instructions, using the recommended cut-off at 14%. This kit was the only commercial kit available at the time for testing SARS-CoV antibodies in a species-independent manner.

### Plasmids, recombinant proteins, and RBDs immunized rabbit sera

Recombinant SARS-CoV-2, SARS-CoV, RaTG13, RmYN02, GX-P5L, and ZC45 RBD proteins with 8X His-tag and AviTag at C-terminal were produced and purified by Genscript (Supplementary Table 6). RBDs immunized rabbit sera were produced by Genscript.

RacCS203 RBD gene was synthesized by Genscript and cloned into pcDNA3.1 expression vector. SARS-CoV-2 spike signaling peptide (aa 1–14) and 10X His tag were fused at the N-terminal and C-terminal, respectively. RacCS203 RBD was produced by transfection of pcDNA3.1 RacCS203-RBD plasmids into HEK293T cells in Opti-MEM (Gibco). Recombinant proteins were harvested at day 3 post-transfection and purified using Ni Sepharose beads (GE healthcare).

### Luminex assays for antigenic profiling of different RBD proteins

Biotinylated SARS-CoV-2, SARS-CoV, RaTG13, RmYN02, GX-P5L, and SL-ZC45 RBD were coated onto MagPlex Avidin microspheres (Luminex) at final concentration of 800 nM in PBS with 1% BSA. Luminex assays were performed by pre-incubation of 1:100 diluted sera with 1250 beads/antigen for 1 h at 37 °C followed by 1:1000 diluted PE-conjugated anti-rabbit IgG (Thermo Scientific) or PE-conjugated anti-human IgG (eBioscience) for 1 h at 37 °C. The level of antibody binding was determined using Luminex MAGPIX system. The readings were normalized against readings obtained from anti-6XHis tag antibody (at final dilution of 1:600) (Thermo Scientific). Panel of COVID-19 and SARS sera used in this study were from Singapore PROTECT study and SARS recalled sampling in 2020 (ethics approval number: NHG DSRB E 2020/00091), both are approved by National Health Group Singapore, with written informed consent for both the use and reporting in the research.

### Multiplex RBD-ACE2 binding assay

RBD proteins from SARS-CoV-2, SARS-CoV, RaTG13, GX-P5L, RmYN02, SL-ZC45, and RacCS203 were all conjugated onto MagPlex-c microsphere (Luminex) using xMAP Antibody Coupling Kit (Luminex) according to the manufacturer’s instructions. To assess ACE2 binding reactivity, 1250 beads/antigen were pre-incubated with PE-conjugated ACE2 (Genscript) for 1 h at 37 °C. The level of ACE2 binding was determined using Luminex MAGPIX system.

### Bat distribution plot

*Rhinolophus* genus bat distribution data across the world (Fig. 4a, b and Supplementary Fig. 4a, b) were derived from spatial data on terrestrial mammals from the International Union for the Conservation of Nature (IUCN 2020. The IUCN Red List of Threatened Species. January 2019 [version 6.2]. https://www.iucnredlist.org; Downloaded on 25 October 2020) and exported into R (version 4.0.2). For the five specific species, the data were extracted with R package “tidyverse” (version 1.3.0). World map data were retrieved and cropped using the R package “rnaturalearth” (version 0.1.0) and “sf” (version 0.9-6). Distribution data were plotted on the cropped world map using r package “ggplot2” (3.3.2). Written permission from IUCN Red List was obtained for the publication of spatial data used in this study.

### Reporting summary

Further information on research design is available in the Nature Research Reporting Summary linked to this article.

## Supplementary information

Supplementary Information

Reporting Summary

## Data Availability

Genome sequences reported in this study have been deposited in GenBank under the accession numbers MW251308, MW251309, MW251310, MW251311 and MW251312. Raw sequencing reads reported in this study have been uploaded into SRA under BioProject ID PRJNA689713. Additional datasets generated and/or analyzed during the current study are available from the corresponding author on reasonable request. Source data are provided with this paper.

## Source data

Source Data
