# Author Correction: Evidence for SARS-CoV-2 related coronaviruses circulating in bats and pangolins in Southeast Asia

**DOI:** 10.1038/s41467-021-21768-2

**Published:** 2021-02-25

**Authors:** Supaporn Wacharapluesadee, Chee Wah Tan, Patarapol Maneeorn, Prateep Duengkae, Feng Zhu, Yutthana Joyjinda, Thongchai Kaewpom, Wan Ni Chia, Weenassarin Ampoot, Beng Lee Lim, Kanthita Worachotsueptrakun, Vivian Chih-Wei Chen, Nutthinee Sirichan, Chanida Ruchisrisarod, Apaporn Rodpan, Kirana Noradechanon, Thanawadee Phaichana, Niran Jantarat, Boonchu Thongnumchaima, Changchun Tu, Gary Crameri, Martha M. Stokes, Thiravat Hemachudha, Lin-Fa Wang

**Affiliations:** 1 Thai Red Cross Emerging Infectious Diseases Health Science Centre, WHO Collaborating Centre for Research and Training on Viral Zoonoses, King Chulalongkorn Memorial Hospital, Faculty of Medicine, Chulalongkorn University, Bangkok, Thailand; 2 Programme in Emerging Infectious Diseases, Duke-NUS Medical School, Singapore, Singapore; 3 Department of National Parks, Wildlife and Plant Conservation, Ministry of Natural Resources and Environment, Bangkok, Thailand; 4 Forest Biology Department, Faculty of Forestry, Kasetsart University, Bangkok, Thailand; 5 Changchun Veterinary Research Institute, Chinese Academy of Agricultural Sciences, Changchun, China; 6 Jiangsu Co-innovation Center for Prevention and Control of Important Animal Infectious Diseases and Zoonosis, Yangzhou University, Yangzhou, China; 7 CSIRO Australian Animal Health Laboratory, Geelong, VIC Australia; 8 Biological Threat Reduction Program, Defense Threat Reduction Agency, Fort Belvoir, VA USA; 9 SingHealth Duke-NUS Global Health Institute, Singapore, Singapore

**Keywords:** Pathogens, SARS-CoV-2

Correction to: *Nature Communications* 10.1038/s41467-021-21240-1, published online 09 February 2021.

The original version of this Article contained an error in the author affiliation.

Lin-Fa Wang was incorrectly associated with Thai Red Cross Emerging Infectious Diseases Health Science Centre, WHO Collaborating Centre for Research and Training on Viral Zoonoses, King Chulalongkorn Memorial Hospital, Faculty of Medicine, Chulalongkorn University, Bangkok, Thailand.

This has now been corrected in both the PDF and HTML versions of the Article.

