# Supplementary material for: Evidence for SARS-CoV-2 related coronaviruses circulating in bats and pangolins in Southeast Asia
Source: Nat Commun. 2021 Feb 9;12:972. doi: 10.1038/s41467-021-21240-1 (PMC7873279; doi:10.1038/s41467-021-21240-1)
Supplement: Supplementary file 1 — Supplementary Information [file 41467_2021_21240_MOESM1_ESM.pdf]

Supplementary figures and tables

for

Evidence for SARS-CoV-2 related coronaviruses circulating in bats and pangolins in Southeast Asia

Supaporn Wacharapluesadee[1]†, Chee Wah Tan[2]†, Patarapol Maneeorn[3]†, Prateep Duengkae[4], Feng Zhu[2], Yutthana Joyjinda[1], Thongchai Kaewpom[1], Wan Ni Chia[2], Weenassarin Ampoot[1], Beng Lee Lim[2], Kanthita Worachotsueptrakun[1], Vivian Chih-Wei Chen[2], Nutthinee Sirichan[4], Chanida Ruchisrisarod[1], Apaporn Rodpan[1], Kirana Noradechanon[3], Thanawadee Phaichana[3], Niran Jantarat[3], Boonchu Thongnumchaima[3], Changchun Tu[5,6], Gary Crameri[7], Martha M. Stokes[8], Thiravat Hemachudha[1*], Lin-Fa Wang[1,9*]

[1]Thai Red Cross Emerging Infectious Diseases Health Science Centre, WHO Collaborating Centre for Research and Training on Viral Zoonoses, King Chulalongkorn Memorial Hospital

Faculty of Medicine, Chulalongkorn University, Thailand

[2]Programme in Emerging Infectious Diseases, Duke-NUS Medical School, Singapore

[3]Department of National Parks, Wildlife and Plant Conservation, Ministry of Natural Resources and Environment, Thailand

[4]Forest Biology Department, Faculty of Forestry, Kasetsart University, Thailand

[5]Changchun Veterinary Research Institute, Chinese Academy of Agricultural Sciences, Changchun, China

[6]Jiangsu Co-innovation Center for Prevention and Control of Important Animal Infectious Diseases and Zoonosis, Yangzhou University, Yangzhou, China

[7]CSIRO Australian Animal Health Laboratory, Geelong, Australia

[8]Biological Threat Reduction Program, Defense Threat Reduction Agency, USA

[9]SingHealth Duke-NUS Global Health Institute, Singapore

†These authors contributed equally

* These authors jointly supervised this work. Correspondence may be addressed to L-FW linfa.wang@duke-nus.edu.sg and TH fmedthm@gmail.com

a

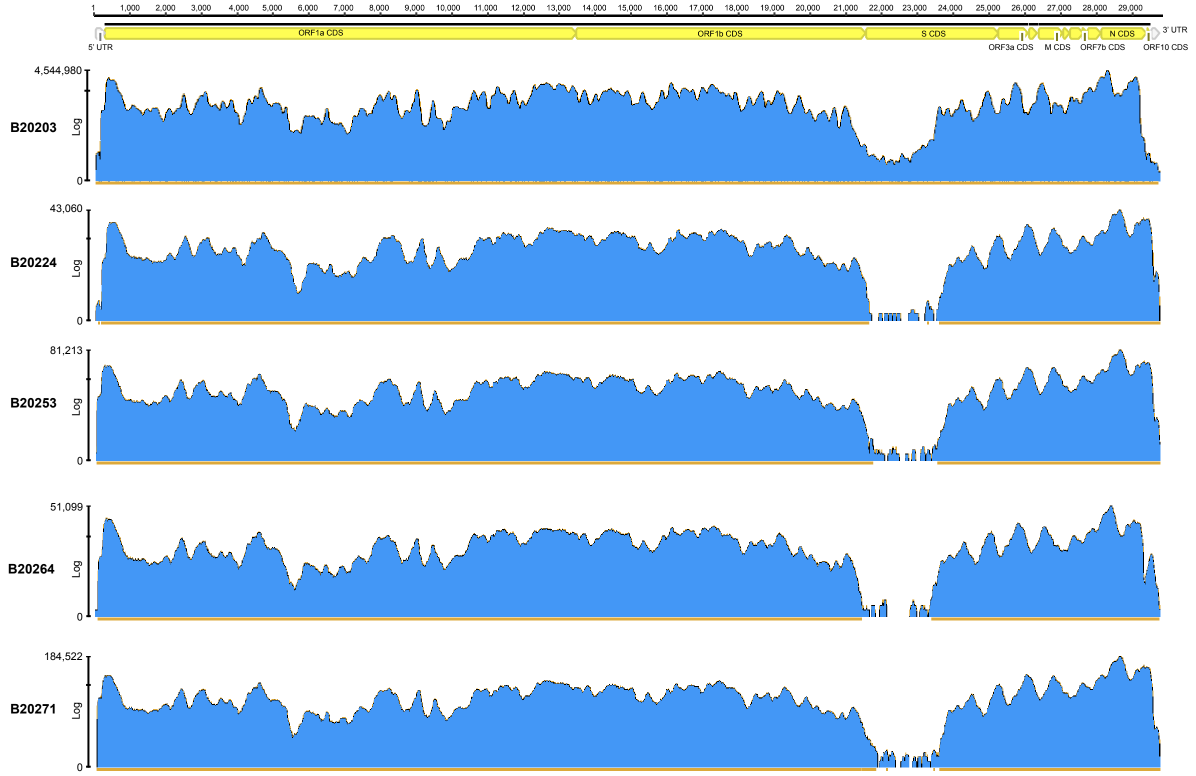

b

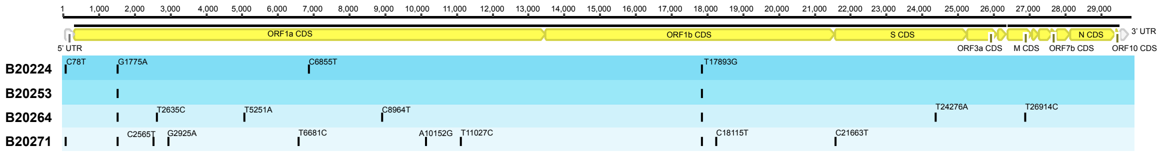

Supplementary Fig. 1| Genome characterization. (a) Quality of the five individual genome assebly, reads coverage are shown on a log scale. Region of coverage lower than 5 reads was marked in orange; (b) SNIP analysis of four genomes mapping onto the reference genome RacCS203. Maximum p-value is 1 x 10^{-6} and minimum variant frequency is 0.25. Low coverage region was marked in rectangle for reference.

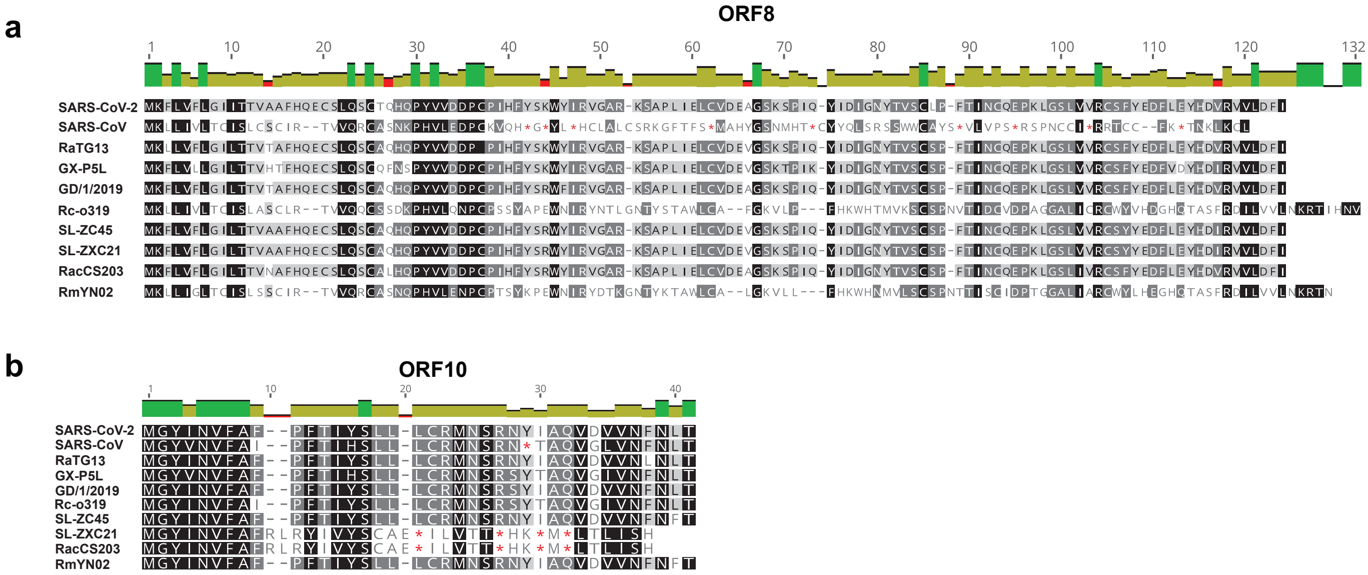

Supplementary Fig. 2| Amino acid sequence comparison. Molecular characterization of (a) ORF8 and (b) ORF10 of RacCS203 and representative SC2r-CoV and SC1r-CoVs. Identity plot was presented above the amino acid alignment. Green indicates high identity and red indicates low identity.

a

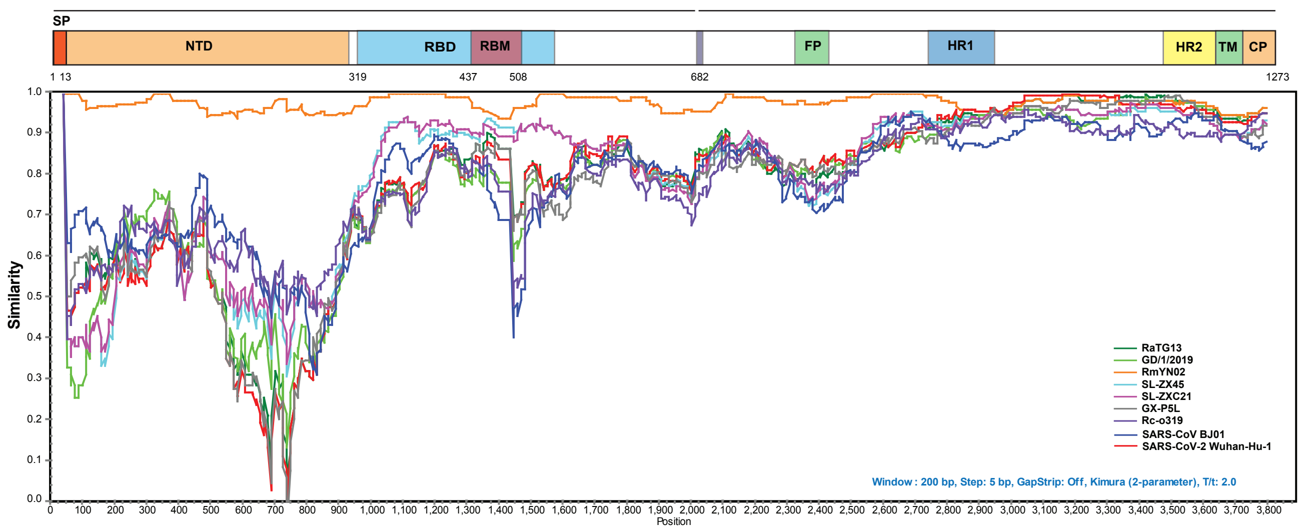

b

Furin cleavage site

```
            671                                    691
SARS-CoV-2  C A S Y Q T Q T N S P R R A R S V - A S Q S
SARS-CoV    C A S Y H T V S L L - - - - R S T - S Q K S
RaTG13      C A S Y Q T Q T N S - - - - R S V - A S Q S
GX-P5L      C A S Y H S M S S F - - - - R S V - N Q R S
GD/1/2019   C A S Y Q T Q T N S - - - - R S V - S S Q A
Rc-o319     C A T Y H T P S M L - - - - R S A N N N K R
SL-ZC45     C A S Y H T A S I L - - - - R S T - S Q K A
SL-ZC45     C A S Y H T A S I L - - - - R S T - G Q K A
RacCS203    C A S Y - - - - N S P - V A R - V - G T N S
RmYN02      C A S Y - - - - N S P - A A R - V - G T N S
```

c **d**

RBD

```
Consensus  RXLPSTEVVRFPNITNFCPFDKVFNATRFPNVYAWQRTKISDCIADYTVLYNSTSFSTFK   60
RacCS203   RVLPSTEVVRFPNITNFCPFDKVFNATRFPNVYAWQRTKISDCIADYTVLYNSTSFSTFK   60
RmYN02     RILPSTEVVRFPNITNFCPFDKVFNATRFPNVYAWQRTKISDCIADYTVLYNSTSFSTFK   60

Consensus  CYGVSPSKLIDLCFTSVYADTFLIRFSEVRQIAPGETGVIADYNYKLPDDFTGCVLAWNT  120
RacCS203   CYGVSPSKLIDLCFTSVYADTFLIRFSEVRQIAPGETGVIADYNYKLPDDFTGCVLAWNT  120
RmYN02     CYGVSPSKLIDLCFTSVYADTFLIRFSEVRQIAPGETGVIADYNYKLPDDFTGCVLAWNT  120

Consensus  AXQDIGSYFYRSHRAVKLKPFERDLSSDENGVRTLSTYDFNPNVPLDYQATRVVVLSFEL  180
RacCS203   AKQDIGSYFYRSHRAVKLKPFERDLSSDENGVRTLSTYDFNPNVPLDYQATRVVVLSFEL  180
RmYN02     AQQDIGSYFYRSHRAVKLKPFERDLSSDENGVRTLSTYDFNPNVPLDYQATRVVVLSFEL  180

Consensus  LNAPATVCGPKLSTQLVKNRCVNF                                      204
RacCS203   LNAPATVCGPKLSTQLVKNRCVNF                                      204
RmYN02     LNAPATVCGPKLSTQLVKNRCVNF                                      204
```

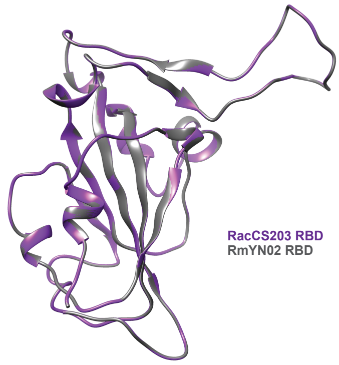

Supplementary Fig. 3| Detailed sequence comparison for S protein. (a) Similarity plot of SC2r-CoV; (b) Multiple alignment of SARSr-CoV at the furin cleavage site; (c) RBD alignment of RacCS203 and RmYN02; (d) RBD modeling to illustrate the key difference between RacCS203 and SARS-CoV-2 using SARS-CoV-2 RBD (PDB: 6VW1) as a template.

a World distribution of *Rhinolophus* species

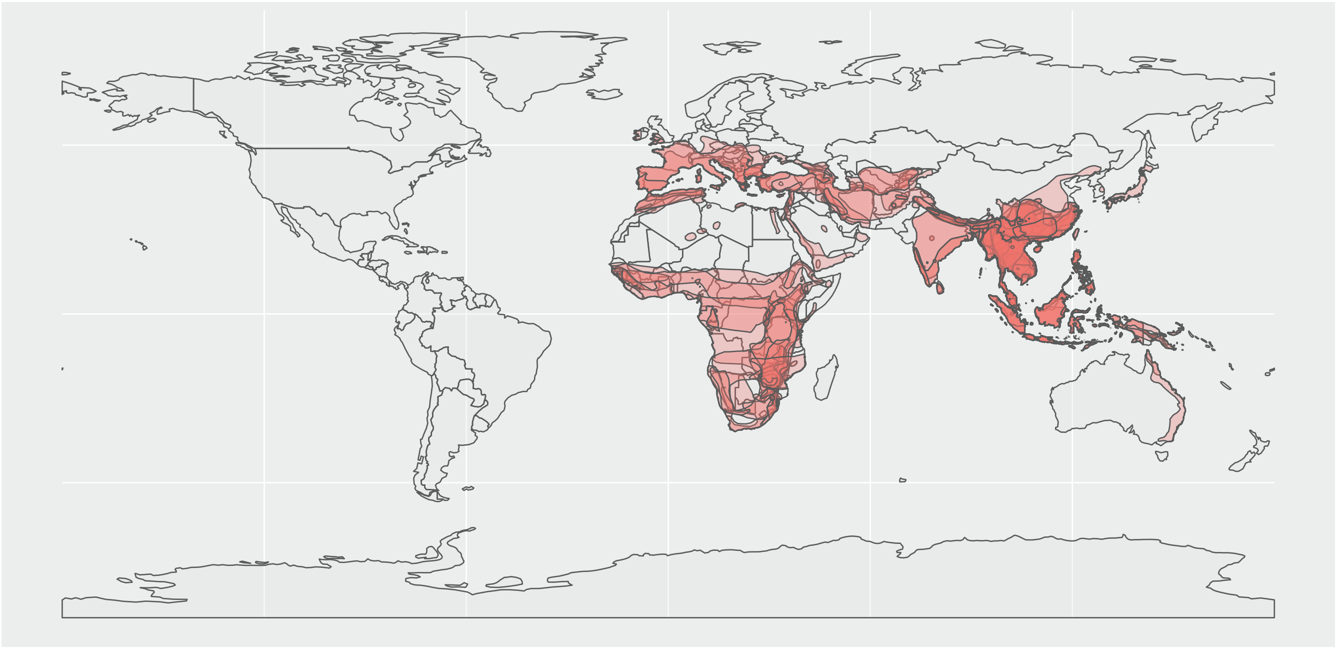

b 5 *Rhinolophus* species Distribution in East and South East Asia

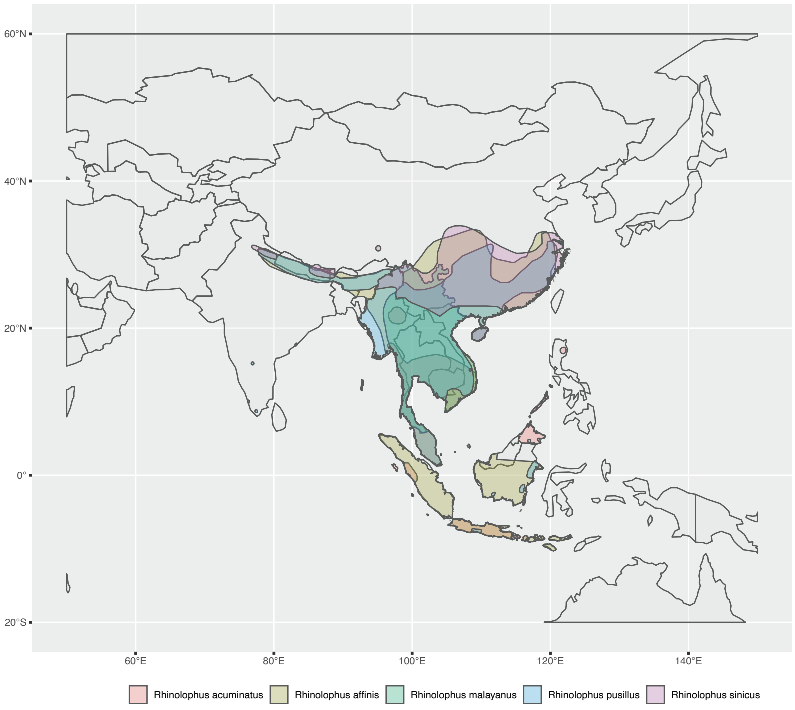

Supplementary Fig. 4| Distribution of *Rhinolophus* bats. (a) World-wide distribution for all 92 *Rhinolophus* species; (b) East and South East Asia distribution of 5 species that are found to contain SC2r-CoVs. Data retrieved from IUCN Red List of Threatened Species version 1.18 https://www.iucnredlist.org. Downloaded on 25th Oct 2020.

Supplementary Table 1. Summary of bat surveillance data

Sample ID #	Collection Date	sVNT	PCR*	Date of Analysis	NGS Analysis
B20201	19/06/2020	<20%	-ve	27/06/2020	
B20202	19/06/2020	<20%	-ve	27/06/2020	
B20203	19/06/2020	<20%	+ve	27/06/2020	Yes
B20204	19/06/2020	<20%	-ve	27/06/2020	
B20205	19/06/2020	<20%	-ve	27/06/2020	
B20206	19/06/2020	<20%	-ve	27/06/2020	
B20207	19/06/2020	<20%	-ve	27/06/2020	
B20208	19/06/2020	<20%	-ve	27/06/2020	
B20209	19/06/2020	<20%	+ve	27/06/2020	
B20210	19/06/2020	<20%	-ve	27/06/2020	
B20211	19/06/2020	<20%	-ve	27/06/2020	
B20212	19/06/2020	<20%	-ve	27/06/2020	
B20213	19/06/2020	34.6%	+ve	27/06/2020	
B20214	19/06/2020	<20%	-ve	27/06/2020	
B20215	19/06/2020	<20%	-ve	27/06/2020	
B20216	19/06/2020	<20%	-ve	27/06/2020	
B20217	19/06/2020	<20%	-ve	27/06/2020	
B20218	19/06/2020	NA†	-ve	27/06/2020	
B20219	19/06/2020	<20%	-ve	27/06/2020	
B20220	19/06/2020	NA	-ve	27/06/2020	
B20221	19/06/2020	<20%	-ve	27/06/2020	
B20222	19/06/2020	<20%	-ve	27/06/2020	
B20223	19/06/2020	<20%	-ve	27/06/2020	
B20224	19/06/2020	<20%	+ve	27/06/2020	Yes
B20225	19/06/2020	<20%	-ve	27/06/2020	
B20226	19/06/2020	<20%	-ve	27/06/2020	
B20227	19/06/2020	<20%	-ve	27/06/2020	
B20228	19/06/2020	<20%	+ve	27/06/2020	
B20229	19/06/2020	<20%	-ve	27/06/2020	
B20230	19/06/2020	<20%	+ve	27/06/2020	
B20231	19/06/2020	<20%	-ve	27/06/2020	
B20232	19/06/2020	<20%	-ve	27/06/2020	
B20233	19/06/2020	<20%	-ve	27/06/2020	
B20234	19/06/2020	<20%	-ve	27/06/2020	
B20235	19/06/2020	<20%	-ve	27/06/2020	
B20236	19/06/2020	<20%	-ve	27/06/2020	
B20237	19/06/2020	<20%	-ve	27/06/2020	
B20238	19/06/2020	<20%	-ve	27/06/2020	
B20239	19/06/2020	<20%	-ve	27/06/2020	
B20240	19/06/2020	<20%	-ve	27/06/2020	
B20241	19/06/2020	<20%	-ve	27/06/2020	
B20242	19/06/2020	<20%	-ve	27/06/2020	
B20243	19/06/2020	<20%	-ve	27/06/2020	
B20244	19/06/2020	<20%	-ve	27/06/2020	
B20245	19/06/2020	<20%	-ve	27/06/2020	
B20246	19/06/2020	<20%	-ve	27/06/2020	
B20247	19/06/2020	<20%	-ve	27/06/2020	
B20248	19/06/2020	<20%	-ve	27/06/2020	
B20249	19/06/2020	<20%	-ve	27/06/2020	

B20250	19/06/2020	<20%	-ve	27/06/2020	
B20251	19/06/2020	<20%	-ve	27/06/2020	
B20252	19/06/2020	<20%	-ve	27/06/2020	
B20253	19/06/2020	36.2%	+ve	27/06/2020	Yes
B20254	19/06/2020	<20%	-ve	27/06/2020	
B20255	19/06/2020	<20%	-ve	27/06/2020	
B20256	19/06/2020	<20%	-ve	27/06/2020	
B20257	19/06/2020	<20%	-ve	27/06/2020	
B20258	19/06/2020	<20%	-ve	27/06/2020	
B20259	19/06/2020	<20%	-ve	27/06/2020	
B20260	19/06/2020	<20%	-ve	27/06/2020	
B20261	19/06/2020	<20%	-ve	27/06/2020	
B20262	20/06/2020	<20%	-ve	27/06/2020	
B20263	20/06/2020	<20%	-ve	27/06/2020	
B20264	20/06/2020	<20%	+ve	27/06/2020	Yes
B20265	20/06/2020	<20%	-ve	27/06/2020	
B20266	20/06/2020	<20%	-ve	27/06/2020	
B20267	20/06/2020	<20%	+ve	27/06/2020	
B20268	20/06/2020	<20%	-ve	27/06/2020	
B20269	20/06/2020	<20%	-ve	27/06/2020	
B20270	20/06/2020	<20%	-ve	27/06/2020	
B20271	20/06/2020	<20%	+ve	27/06/2020	Yes
B20272	20/06/2020	94.7%	+ve	27/06/2020	
B20273	20/06/2020	<20%	-ve	08/07/2020	
B20274	20/06/2020	<20%	-ve	08/07/2020	
B20275	20/06/2020	<20%	-ve	08/07/2020	
B20276	20/06/2020	<20%	-ve	08/07/2020	
B20277	20/06/2020	<20%	-ve	08/07/2020	
B20278	20/06/2020	<20%	+ve	08/07/2020	
B20279	20/06/2020	<20%	-ve	08/07/2020	
B20280	20/06/2020	<20%	-ve	08/07/2020	
B20281	20/06/2020	<20%	-ve	08/07/2020	
B20282	20/06/2020	<20%	-ve	08/07/2020	
B20283	20/06/2020	<20%	-ve	08/07/2020	
B20284	20/06/2020	<20%	-ve	08/07/2020	
B20285	20/06/2020	<20%	-ve	08/07/2020	
B20286	20/06/2020	<20%	-ve	08/07/2020	
B20287	20/06/2020	<20%	-ve	08/07/2020	
B20288	20/06/2020	<20%	-ve	08/07/2020	
B20289	20/06/2020	<20%	-ve	08/07/2020	
B20290	20/06/2020	87.4%	+ve	08/07/2020	
B20291	20/06/2020	<20%	-ve	08/07/2020	
B20292	20/06/2020	<20%	-ve	08/07/2020	
B20293	20/06/2020	<20%	-ve	08/07/2020	
B20294	20/06/2020	<20%	-ve	08/07/2020	
B20295	20/06/2020	<20%	-ve	08/07/2020	
B20296	20/06/2020	<20%	-ve	08/07/2020	
B20297	20/06/2020	<20%	-ve	08/07/2020	
B20298	20/06/2020	<20%	-ve	08/07/2020	
B20299	20/06/2020	<20%	-ve	08/07/2020	
B20300	20/06/2020	<20%	-ve	08/07/2020	

† NA = sample not available

Supplementary Table 2. Sequence comparison of ortholog genes and proteins between SARS-CoV-2 and other SARSr-CoVs

	**Strain**	**Complete genome**	**Gene region**											
**Nucleotide**			**1ab**	**S**	**RBD**	**3a**	**E**	**M**	**6**	**7a**	**7b**	**8**	**N**	**10**
	**RacCS203**	91.5%	94.3%	71.3%	61.6%	91.9%	99.1%	94.6%	96.2%	92.4%	93.9%	91.6%	93.2%	99.2%
	**RmYN02**	93.6%	97.1%	72.5%	61.9%	96.4%	98.7%	94.8%	96.8%	96.2%	91.0%	48.7%	97.3%	99.2%
	**SL-ZC45**	87.7%	89.0%	75.5%	62.5%	87.8%	98.7%	93.4%	94.6%	88.8%	94.7%	88.5%	91.1%	99.2%
	**SL-ZXC21**	87.5%	88.7%	74.9%	61.6%	88.9%	98.7%	93.4%	94.6%	89.1%	95.5%	88.5%	91.2%	99.2%
	**Rc-o319**	79.2%	79.8%	72.2%	70.1%	83.3%	97.4%	86.6%	86.6%	78.4%	77.3%	52.3%	88.3%	94.9%
	**GD/1/2019**	90.2%	90.2%	83.7%	86.9%	93.2%	99.1%	93.3%	95.7%	93.4%	91.7%	92.1%	96.2%	99.2%
	**GX-P5L**	85.2%	84.6%	83.3%	79.9%	87.0%	97.4%	91.3%	90.9%	86.4%	83.5%	80.7%	91.0%	94.0%
	**GX-P4L**	85.4%	84.8%	83.6%	80.0%	86.8%	97.4%	91.3%	90.9%	86.6%	83.5%	81.3%	91.0%	88.9%
	**RaTG13**	96.1%	96.5%	92.3%	86.3%	96.3%	99.6%	95.5%	98.4%	95.6%	99.2%	97.0%	96.9%	99.2%
	**SARS-CoV**	79.3%	79.7%	72.3%	71.9%	75.3%	93.5%	85.5%	75.5%	82.1%	83.8%	45.8%	88.2%	93.2%
**Amino acid**			**1ab**	**S**	**RBD**	**3a**	**E**	**M**	**6**	**7a**	**7b**	**8**	**N**	**10**
	**RacCS203**		97.3%	72.3%	63.7%	97.5%	100.0%	99.1%	98.4%	95.9%	93.0%	94.2%	95.7%	-
	**RmYN02**		98.8%	72.4%	63.2%	96.7%	100.0%	98.7%	96.7%	95.9%	83.7%	28.2%	98.6%	97.4%
	**SL-ZC45**		95.6%	80.2%	65.9%	90.9%	100.0%	98.7%	93.4%	87.6%	93.0%	94.2%	94.3%	97.4%
	**SL-ZXC21**		95.2%	79.7%	65.9%	92.0%	100.0%	98.7%	93.4%	88.4%	93.0%	94.2%	94.3%	-
	**Rc-o319**		87.6%	76.2%	73.5%	87.0%	98.7%	91.0%	83.6%	73.8%	69.8%	26.8%	89.5%	86.8%
	**GD/1/2019**		96.7%	90.0%	96.9%	97.1%	100.0%	98.7%	96.7%	97.5%	95.4%	95.0%	97.9%	97.4%
	**GX-P5L**		92.5%	92.4%	86.6%	89.8%	100.0%	98.2%	95.1%	88.4%	72.1%	87.6%	93.8%	84.2%
	**GX-P4L**		92.5%	92.3%	86.6%	89.5%	100.0%	98.2%	95.1%	88.4%	-	87.6%	93.6%	73.7%
	**RaTG13**		98.5%	97.3%	90.1%	97.8%	100.0%	99.6%	100.0%	97.5%	97.7%	95.0%	99.1%	97.4%
	**SARS-CoV**		86.1%	75.8%	73.1%	72.4%	94.7%	90.5%	67.2%	85.3%	81.4%	-	90.5%	81.6%

-indicates ORF with incomplete cds or early termination.

Supplementary Table 3: Sequence comparison of ortholog genes and proteins between RacCS203 and other SARSr-CoVs

	**Strain**	**Complete genome**	**Gene region**											
**Nucleotide**			**1ab**	**S**	**RBD**	**3a**	**E**	**M**	**6**	**7a**	**7b**	**8**	**N**	**10**
	**SARS-CoV-2**	91.5%	94.3%	71.3%	61.6%	91.9%	99.1%	94.3%	96.2%	92.4%	93.9%	91.6%	93.2%	99.2%
	**RmYN02**	93.7%	94.3%	94.3%	95.4%	92.0%	97.8%	94.1%	95.2%	91.5%	87.3%	48.2%	93.7%	98.3%
	**SL-ZC45**	86.7%	88.0%	73.5%	78.6%	86.4%	97.8%	92.8%	92.5%	88.4%	93.2%	88.0%	92.5%	98.3%
	**SL-ZXC21**	86.6%	87.8%	73.8%	79.4%	87.6%	97.8%	92.8%	92.5%	88.8%	93.9%	88.0%	92.5%	100.0%
	**Rc-o319**	78.3%	79.8%	69.8%	61.8%	82.5%	96.5%	86.9%	85.5%	76.0%	75.0%	52.0%	86.0%	94.9%
	**GD/1/2019**	88.0%	90.2%	70.6%	61.0%	90.9%	98.3%	93.4%	95.2%	90.4%	90.2%	91.8%	93.4%	99.2%
	**GX-P5L**	83.1%	84.2%	70.8%	64.1%	87.0%	96.5%	92.1%	89.3%	83.1%	82.7%	80.4%	90.0%	93.2%
	**GX-P4L**	83.7%	84.4%	72.9%	67.0%	87.1%	96.5%	92.1%	89.3%	83.3%	83.3%	80.3%	90.0%	88.0%
	**RaTG13**	91.4%	93.9%	71.7%	62.5%	92.0%	98.7%	94.5%	94.6%	92.9%	94.7%	92.1%	93.9%	98.3%
	**SARS-CoV**	78.4%	79.5%	69.3%	64.2%	75.0%	93.5%	85.2%	76.3%	82.1%	980.2%	45.5%	88.6%	93.2%
**Amino acid**			**1ab**	**S**	**RBD**	**3a**	**E**	**M**	**6**	**7a**	**7b**	**8**	**N**	**10**
	**SARS-CoV-2**		97.3%	72.2%	63.7%	97.5%	100%	99.10%	98.4%	95.9%	93.0%	94.2%	95.7%	-
	**RmYN02**		97.2%	98.0%	99.0%	96.7%	100%	98.6%	98.4%	95.0%	81.4%	29.8%	95.7%	-
	**SL-ZC45**		94.7%	76.9%	90.2%	91.3%	100%	98.2%	95.1%	87.6%	95.4%	95.0%	95.7%	-
	**SL-ZXC21**		94.2%	77.1%	89.8%	92.4%	100%	98.2%	95.1%	88.4%	95.4%	95.0%	95.7%	-
	**Rc-o319**		87.2%	75.0%	64.5%	86.6%	98.7%	90.5%	82.0%	74.6%	67.4%	27.6%	88.3%	-
	**GD/1/2019**		96.2%	73.1%	63.7%	97.5%	100%	98.6%	98.4%	95.0%	97.7%	96.7%	95.0%	-
	**GX-P5L**		92.1%	73.0%	64.1%	90.6%	100%	99.6%	96.7%	86.0%	72.1%	87.6%	93.6%	-
	**GX-P4L**		92.1%	73.3%	64.1%	90.2%	100%	99.6%	96.7%	85.6%	-	87.6%	93.6%	-
	**RaTG13**		97.1%	72.4%	62.8%	97.5%	100%	99.6%	98.4%	95.0%	95.4%	95.0%	95.7%	-
	**SARS-CoV**		86.1%	71.7%	64.0%	72.0%	94.7%	91.0%	68.9%	83.6%	76.7%	-	91.5%	-

-indicates ORF with incomplete cds or early termination.

Supplementary Table 4. Pan-CoV primers used in this study

**Primer name**	**Sequence (5' to 3')**	**PCR condition**
CoV-FWD1	CGTTGGIACWAAYBTVCCWYTICARBTRGG	95°C for 5 min;<br>15 cycles of 95°C for 30s, 65°C for 30s and 72°C for 45s;<br>35 cycles of 95°C for 30s, 50°C for 30s and 72°C for 45s;<br>72°C for 7 min.
CoV-RVS1	GGTCATKATAGCRTCAVMASWWGCNACNACATG	
CoV-FWD2	GGCWCCWCCHGGNGARCAATT	95°C for 5 min;<br>15 cycles of 95°C for 30s, 65°C for 30s and 72°C for 45s;<br>35 cycles of 95°C for 30s, 50°C for 30s and 72°C for 45s;<br>72°C for 7 min.
CoV-RVS2	GGWAWCCCCAYTGYTGWAYRTC	

Supplementary Table 5. Accession numbers of the SARSr-CoVs used in this study

Accession number	SARSr-CoV	Host
AY278488	SARS coronavirus BJ01	*Homo sapiens*
DQ022305	Bat SC1r-CoV HKU3-1	*Rhinolophus sinicus*
DQ071615	Bat SC1r-CoV Rp3	*Rhinolophus sinicus, Rhinolophus ferrumequinum*
DQ412042	Bat SC1r-CoV Rf1	*Rhinolophus ferrumequinum*
DQ412043	Bat SC1r-CoV Rm1	*Rhinolophus macrotis*
DQ648856	Bat SC1r-CoV 273/2005	*Rhinolophus ferrumequinum*
DQ648857	Bat SC1r-CoV 279/2005	*Rhinolophus macrotis*
FJ588686	Bat SC1r-CoV Rs672/2006	*Rhinolophus sinicus*
EPI_ISL_410721	Pangolin SC2r-CoV GD/1/2019	*Manis javanica*
EPI_ISL_412977	Bat SC2r-CoV RmYN02	*Rhinolophus malayanus*
JX993987	Bat SC1r-CoV Rp/Shaanxi2011	*Rhinolophus pusillus*
JX993988	Bat SC1r-CoV Cp/Yunnan2011	*Chaerephon plicata*
KC881005	Bat SC1r-CoV RsSHC014	*Rhinolophus sinicus*
KC881006	Bat SC1r-CoV Rs3367	*Rhinolophus sinicus*
KF294457	Bat SC1r-CoV Longquan-140	*Rhinolophus monoceros*
KF367457	Bat SC1r-CoV WIV1	*Rhinolophus sinicus*
KF569996	Bat SC1r-CoV LYRa11	*Rhinolophus affinis*
KJ473811	Bat SC1r-CoV JL201	*Rhinolophus ferrumequinum*
KJ473812	Bat SC1r-CoV HeB2013	*Rhinolophus ferrumequinum*
KJ473813	Bat SC1r-CoV SX2013	*Rhinolophus ferrumequinum*
KJ473814	Bat SC1r-CoV HuB2013	*Rhinolophus sinicus*
KJ473815	Bat SC1r-CoV GX2013	*Rhinolophus sinicus*
KP886808	Bat SC1r-CoV YNLF_31C	*Rhinolophus ferrumequinum*
KP886809	Bat SC1r-CoV YNLF_34C	*Rhinolophus ferrumequinum*
KU182964	Bat SC1r-CoV JTMC15	*Rhinolophus ferrumequinum*
KY352407	Bat SC1r-CoV BtKY72	*Rhinolophus sp. (bat)*
KY417142	Bat SC1r-CoV As6526	*Aselliscus stoliczkanus*
KY417143	Bat SC1r-CoV Rs4081	*Rhinolophus sinicus*
KY417144	Bat SC1r-CoV Rs4084	*Rhinolophus sinicus*
KY417145	Bat SC1r-CoV Rf4092	*Rhinolophus ferrumequinum*
KY417146	Bat SC1r-CoV Rs4231	*Rhinolophus sinicus*
KY417147	Bat SC1r-CoV Rs4237	*Rhinolophus sinicus*
KY417148	Bat SC1r-CoV Rs4247	*Rhinolophus sinicus*
KY417149	Bat SC1r-CoV Rs4255	*Rhinolophus sinicus*
KY417151	Bat SC1r-CoV Rs7327	*Rhinolophus sinicus*
KY417152	Bat SC1r-CoV Rs9401	*Rhinolophus sinicus*
KY770858	Bat SC1r-CoV Anlong-103	*Rhinolophus sinicus*
KY938558	Bat SC1r-CoV 16BO133	*Rhinolophus ferrumequinum*
LC556375	Bat SC2r-CoV Rc-o319	*Rhinolophus cornutus*
MG772933	Bat SC2r-CoV ZC45	*Rhinolophus pusillus*
MG772934	Bat SC2r-CoV ZXC21	*Rhinolophus pusillus*
MN996532	Bat SC2r-CoV RaTG13	*Rhinolophus affinis*
MT040333	Pangolin SC2r-CoV GX-P4L	*Manis javanica*
MT040335	Pangolin SC2r-CoV GX-P5L	*Manis javanica*
NC_014470	Bat SC1r-CoV BM48-31/BGR/2008	*Rhinolophus blasii*
NC_045512	SARS-CoV-2 Wuhan-Hu-1	*Homo sapiens*
MW251308	Bat SC2r-CoV RacCS203	*Rhinolophus acuminatus*

Supplementary Table 6. Amino acid sequences of the SARSr-CoV RBD proteins used in this study

Protein	Amino acid sequence	Source
**SARS-CoV-2 RBD**	MGWSCIILFLVATATGVHSRVQPTESIVRFPNITNLCPFGEVFNATRFASVYAWNRKRIS<br>NCVADYSVLYNSASFSTFKCYGVSPTKLNDLCFTNVYADSFVIRGDEVRQIAPGQTGKI<br>ADYNYKLPDDFTGCVIAWNSNNLDSKVGGNYNYLYRLFRKSNLKPFERDISTEIYQAG<br>STPCNGVEGFNCYFPLQSYGFQPTNGVGYQPYRVVVLSFELLHAPATVCGPKKSTNLV<br>KNKCVNFNFNGLTGTGVLTESNKKFLPFQQFGRDIADTTDAVRDPQTLEILDITPCSHH<br>HHHHHHGGGSGLNDIFEAQKIEWHE	GenScript
**SARS-CoV RBD**	MGWSCIILFLVATATGVHSNITNLCPFGEVFNATKFPSVYAWERKKISNCVADYSVLYN<br>STFFSTFKCYGVSATKLNDLCFSNVYADSFVVKGDDVRQIAPGQTGVIADYNYKLPDD<br>FMGCVLAWNTRNIDATSTGNYNYKYRYLRHGKLRPFERDISNVPFSPDGKPCTPPALN<br>CYWPLNDYGFYTTTGIGYQPYRVVVLSFELLNAPATYLEHHHHHHHHGGGSGLNDIFE<br>AQKIEWHE	GenScript
**SC2r-CoV RaTG13**	MGWSCIILFLVATATGVHSRVQPTDSIVRFPNITNLCPFGEVFNATTFASVYAWNRKRIS<br>NCVADYSVLYNSTSFSTFKCYGVSPTKLNDLCFTNVYADSFVITGDEVRQIAPGQTGKI<br>ADYNYKLPDDFTGCVIAWNSKHIDAKEGGNFNYLYRLFRKANLKPFERDISTEIYQAG<br>SKPCNGQTGLNCYYPLYRYGFYPTDGVGHQPYRVVVLSFELLNAPATVCGPKKSTNL<br>VKNKCVNFNFNGLTGTGVLTESNKKFLPFQQFGRDIADTTDAVRDPQTLEILDITPCSH<br>HHHHHHHGGGSGLNDIFEAQKIEWHE	GenScript
**SC2r-CoV RmYN02**	MGWSCIILFLVATATGVHSRILPSTEVVRFPNITNFCPFDKVFNATRFPNVYAWQRTKIS<br>DCIADYTVLYNSTSFSTFKCYGVSPSKLIDLCFTSVYADTFLIRFSEVRQIAPGETGVIAD<br>YNYKLPDDFTGCVLAWNTAQQDIGSYFYRSHRAVKLKPFERDLSSDENGVRTLSTYDF<br>NPNVPLDYQATRVVVLSFELLNAPATVCGPKLSTQLVKNRCVNFNFNGLRGTGVLTDS<br>DKRFQSFQQFGRDSADFTDSVRDPQTLQILDISPCSHHHHHHHHGGGSGLNDIFEAQKI<br>EWHE	GenScript
**SC2r-CoV GX-P5L**	MGWSCIILFLVATATGVHSRVQPTISIVRFPNITNLCPFGEVFNASKFASVYAWNRKRIS<br>NCVADYSVLYNSTSFSTFKCYGVSPTKLNDLCFTNVYADSFVVKGDEVRQIAPGQTGV<br>IADYNYKLPDDFTGCVIAWNSVKQDALTGGNYGYLYRLFRKSKLKPFERDISTEIYQA<br>GSTPCNGQVGLNCYYPLERYGFHPTTGVNYQPFRVVVLSFELLNGPATVCGPKLSTTL<br>VKDKCVNFNFNGLTGTGVLTTSKKQFLPFQQFGRDISDTTDAVRDPQTLEILDITPCSH<br>HHHHHHHGGGSGLNDIFEAQKIEWHE	GenScript
**SC2r-CoV ZC45**	MGWSCIILFLVATATGVHSRVQPTQSVVRFPNITNVCPFHKVFNATRFPSVYAWERTKI<br>SDCIADYTVFYNSTSFSTFKCYGVSPSKLIDLCFTSVYADTFLIRFSEVRQVAPGQTGVIA<br>DYNYKLPDDFTGCVIAWNTAKQDVGNYFYRSHRSTKLKPFERDLSSDENGVRTLSTY<br>DFNPNVPLEYQATRVVVLSFELLNAPATVCGPKLSTQLVKNQCVNFNFNGLKGTGVLT<br>DSSKRFQSFQQFGKDASDFIDSVRDPQTLEILDITPCSHHHHHHHHGGGSGLNDIFEAQK<br>IEWHE	GenScript
**SC2r-CoV RacCS203**	MFVFLVLLPLVSSQRVLPSTEVVRFPNITNFCPFDKVFNATRFPNVYAWQRTKISDCIAD<br>YTVLYNSTSFSTFKCYGVSPSKLIDLCFTSVYADTFLIRFSEVRQIAPGETGVIADYNYKL<br>PDDFTGCVLAWNTAKQDIGSYFYRSHRAVKLKPFERDLSSDENGVRTLSTYDFNPNVP<br>LDYQATRVVVLSFELLNAPATVCGPKLSTQLVKNRCVNFNFNGLRGTGVLTDSDKRFQ<br>SFQQFGRDSADFTDSVRDPQTLQILDISPCSGGGSHHHHHHHHH	In-house
