# Supplementary material for: Evidence for SARS-CoV-2 related coronaviruses circulating in bats and pangolins in Southeast Asia
Source: Nat Commun. 2021 Feb 9;12:972. doi: 10.1038/s41467-021-21240-1 (PMC7873279; doi:10.1038/s41467-021-21240-1)
Supplement: Supplementary file 2 — Reporting Summary [file 41467_2021_21240_MOESM2_ESM.pdf]

nature research

Corresponding author(s): Lin-Fa Wang

Last updated by author(s): Jan 11, 2021

Reporting Summary

Nature Research wishes to improve the reproducibility of the work that we publish. This form provides structure for consistency and transparency in reporting. For further information on Nature Research policies, see our Editorial Policies and the Editorial Policy Checklist.

Statistics

For all statistical analyses, confirm that the following items are present in the figure legend, table legend, main text, or Methods section.

n/a	Confirmed	
☐	☒	The exact sample size (*n*) for each experimental group/condition, given as a discrete number and unit of measurement
☐	☒	A statement on whether measurements were taken from distinct samples or whether the same sample was measured repeatedly
☒	☐	The statistical test(s) used AND whether they are one- or two-sided<br>*Only common tests should be described solely by name; describe more complex techniques in the Methods section.*
☒	☐	A description of all covariates tested
☒	☐	A description of any assumptions or corrections, such as tests of normality and adjustment for multiple comparisons
☐	☒	A full description of the statistical parameters including central tendency (e.g. means) or other basic estimates (e.g. regression coefficient) AND variation (e.g. standard deviation) or associated estimates of uncertainty (e.g. confidence intervals)
☒	☐	For null hypothesis testing, the test statistic (e.g. *F*, *t*, *r*) with confidence intervals, effect sizes, degrees of freedom and *P* value noted<br>*Give P values as exact values whenever suitable.*
☒	☐	For Bayesian analysis, information on the choice of priors and Markov chain Monte Carlo settings
☒	☐	For hierarchical and complex designs, identification of the appropriate level for tests and full reporting of outcomes
☒	☐	Estimates of effect sizes (e.g. Cohen's *d*, Pearson's *r*), indicating how they were calculated

Our web collection on statistics for biologists contains articles on many of the points above.

Software and code

Policy information about availability of computer code

Data collection	Reference genome sequence data were downloaded from GenBank and GISAID using the web interface.
Data analysis	GraphPad Prism 8; Geneious Prime 2020.2.4; PhyML3.0, Simplot v3.5.1; R (version 4.0.2); BBDuk v38.84; SPAdes 3.13.0; SWISS-MODEL (https://swissmodel.expasy.org); UCSF Chimera 1.14; R package "tidyverse" (version 1.3.0); R package "rnaturalearth" (version 0.1.0) and "sf" (version 0.9-6); R package "ggplot2" (3.3.2).

For manuscripts utilizing custom algorithms or software that are central to the research but not yet described in published literature, software must be made available to editors and reviewers. We strongly encourage code deposition in a community repository (e.g. GitHub). See the Nature Research guidelines for submitting code & software for further information.

Data

Policy information about availability of data

All manuscripts must include a data availability statement. This statement should provide the following information, where applicable:

- Accession codes, unique identifiers, or web links for publicly available datasets
- A list of figures that have associated raw data
- A description of any restrictions on data availability

Genome sequences reported in this study have been deposited in GenBank under the accession numbers MW251308, MW251309, MW251310, MW251311 and MW251312. Raw sequencing reads reported in this study have been uploaded into SRA under BioProject ID PRJNA689713. Additional datasets generated and/or analysed during the current study are available from the corresponding author on reasonable request.

Field-specific reporting

Please select the one below that is the best fit for your research. If you are not sure, read the appropriate sections before making your selection.

☒ Life sciences ☐ Behavioural & social sciences ☐ Ecological, evolutionary & environmental sciences

For a reference copy of the document with all sections, see nature.com/documents/nr-reporting-summary-flat.pdf

Life sciences study design

All studies must disclose on these points even when the disclosure is negative.

Sample size	100 Rhinolophus acuminatus bats were captured from an artificial cave in Wildlife Sanctuary, Chachoengsao Province. 10 pangolin sampled during February-July 2020 from 3 wildlife checkpoint stations in Central and Southern Thailand. 7 pangolin sampled in May 2003 from Guangdong Wildlife Conservation and Protection Center. We screened all the available bat and pangolin samples during the study period. Of 100 bat samples, 13 bats are positive by pan-CoV PCR with identical nucleotide sequence, shared 96.21% and 95.86% identity to SC2r-CoV RaTG13 and SARS-CoV-2 respectively. Therefore, such sample size is sufficient for the discovery of SC2r-CoV in bats in our conditions.
Data exclusions	No data were excluded
Replication	Surrogate virus neutralization test, PRNT, Luminex assay were performed in duplication. qPCR positive samples with high Ct value (n = 5) were further examined by enrichment library preparation and next generation sequencing. All attempts at replication were successful.
Randomization	There was no separation of experimental groups in the study, hence no randomization.
Blinding	There was no separation of experimental groups in the study. Therefore, the investigators were not blinded during experiments and outcome assessment.

Reporting for specific materials, systems and methods

We require information from authors about some types of materials, experimental systems and methods used in many studies. Here, indicate whether each material, system or method listed is relevant to your study. If you are not sure if a list item applies to your research, read the appropriate section before selecting a response.

Materials & experimental systems

n/a	Involved in the study
☐	☒ Antibodies
☐	☒ Eukaryotic cell lines
☒	☐ Palaeontology and archaeology
☐	☒ Animals and other organisms
☐	☒ Human research participants
☒	☐ Clinical data
☒	☐ Dual use research of concern

Methods

n/a	Involved in the study
☒	☐ ChIP-seq
☒	☐ Flow cytometry
☒	☐ MRI-based neuroimaging

Antibodies

Antibodies used	All antibodies used for luminex are purchased commercially.<br>Goat anti-rabbit IgG (H+L) cross-adsorbed secondary antibody, PE (ThermoFisher Scientific, Catalog # P-2771 MP)<br>Goat anti-huamn IgG Fc secondary antibody, PE (eBioscience, Catalog # 23-4998-82)<br>Anti-6xHis tag antibody (MA1-135, Thermo Scientific, Catalog # MA1-135)<br>RaTG13 or RmYN02 RBD immunized rabbit sera were custom raised by GenScript.
Validation	All the antibodies used in this study were commercial antibodies and were only used for application, with validation procedures described by the manufacturers. The specificity of anti-6X His Tag antibody (MA1-135) to his-tag were validated by Western Blot, ELISA and Immunoprecipitation. Rabbit polyclonal sera raised against RaTG13 or RmYN02 RBD were validated by specific RBD ELISA.

Eukaryotic cell lines

Policy information about cell lines

Cell line source(s)	Vero E6 (ATCC # CRL 1586) and HEK293T (ATCC # CRL-3216).
Authentication	No authentication was performed.

Mycoplasma contamination	We confirm that all cell lines were negative for mycoplasma contamination
Commonly misidentified lines (See ICLAC register)	No commonly misidentified cell line were used.

Animals and other organisms

Policy information about studies involving animals; ARRIVE guidelines recommended for reporting animal research

Laboratory animals	Not applicable
Wild animals	100 Acuminate Horseshoe bats (Rhinolophus acuminatus) were captured from a Wildlife Sanctuary in the Chachoengsao Province, Thailand. Bats were released after measurements and samples were collected. Malayan pangolin (Manis javanica) samples were collected between February and July 2020 from 3 Wildlife Checkpoint stations, Department of National Parks, Wildlife and Plant Conservation in Central and Southern Thailand with unknown country origin and sent to TRC-EID laboratory in Bangkok within 24 h for testing. For the investigation of pangolins in China, samples were collected from the Guangdong Wildlife Conservation and Protection Center with special permit issued by the National SARS Research Coordination Committee.
Field-collected samples	Rectal swab, blood (50 uL) and wing tissue were collected from individual bat. Bats were free immediately after sample collection. Bat species was identified by morphology and confirmed by mitochrondia DNA. 2-3 mL Blood was collected from pangolin.
Ethics oversight	Sampling was performed under protocols approved and permitted by the Department of National Parks, Wildlife and Plant Conservation, Thailand (No. 0909.204/2686) and the Animal Use Protocol No.1473001 approved by Chulalongkorn University Animal Care and Use Committee.

Note that full information on the approval of the study protocol must also be provided in the manuscript.

Human research participants

Policy information about studies involving human research participants

Population characteristics	Convalescence plasma/serum was collected from patient with confirmed SARS-COV-2 or SARS-CoV infection. All patient information (Age, gender, disease severity etc) was deidentified.
Recruitment	COVID-19 patient recruited under Singapore PROTECT study. SARS survivor recruited under SARS-2020 sampling study (NHG DSRB E 2020/00091). All recruitment was conducted by staff members at the National Center for Infectious Diseases, Singapore. No selection process was involved as all PCR-confirmed COVID-19 patients were recruited for this study as part of a nation-wide COVID-19 response in Singapore.
Ethics oversight	Ethics oversight for laboratory work covered by ethics committees of the Duke-NUS Medical School and National University of Singapore.

Note that full information on the approval of the study protocol must also be provided in the manuscript.
